# High Hydrostatic Pressure-Based Combination Strategies for Microbial Inactivation of Food Products: The Cases of Emerging Combination Patterns

**DOI:** 10.3389/fnut.2022.878904

**Published:** 2022-05-11

**Authors:** Qiang Xia, Qianqian Liu, Gabriela I. Denoya, Caijiao Yang, Francisco J. Barba, Huaning Yu, Xiaojia Chen

**Affiliations:** ^1^State Key Laboratory of Dairy Biotechnology, Shanghai Engineering Research Center of Dairy Biotechnology, Dairy Research Institute, Bright Dairy & Food Co., Ltd., Shanghai, China; ^2^State Key Laboratory of Quality Research in Chinese Medicine, Institute of Chinese Medical Sciences, University of Macau, Macau, Macau SAR, China; ^3^Institute of Environmental Research at Greater Bay Area, Guangzhou University, Guangzhou, China; ^4^Instituto Nacional de Tecnología Agropecuaria (INTA), Instituto Tecnología de Alimentos, Buenos Aires, Argentina; ^5^Instituto de Ciencia y Tecnología de Sistemas Alimentarios Sustentables, UEDD INTA CONICET, Buenos Aires, Argentina; ^6^Consejo Nacional de Investigaciones Científicas y Técnicas (CONICET), Buenos Aires, Argentina; ^7^Key Laboratory of Animal Protein Food Processing Technology of Zhejiang Province, College of Food and Pharmaceutical Sciences, Ningbo University, Ningbo, China; ^8^Nutrition and Food Science Area, Preventive Medicine and Public Health, Food Sciences, Toxicology and Forensic Medicine Department, Faculty of Pharmacy, Universitat de València, Valencia, Spain

**Keywords:** high hydrostatic pressure, plant-based foods, process development and integration, inactivation efficiencies, combined techniques

## Abstract

The high demand for fresh-like characteristics of vegetables and fruits (V&F) boosts the industrial implementation of high hydrostatic pressure (HHP), due to its capability to simultaneously maintain original organoleptic characteristics and to achieve preservative effect of the food. However, there remains great challenges for assuring complete microbial inactivation only relying on individual HHP treatments, including pressure-resistant strains and regrowth of injured microbes during the storage process. Traditional HHP-assisted thermal processing may compromise the nutrition and functionalities due to accelerated chemical kinetics under high pressure conditions. This work summarizes the recent advances in HHP-based combination strategies for microbial safety, as exemplified by several emerging non-thermally combined patterns with high inactivation efficiencies. Considerations and requirements about future process design and development of HHP-based combination technologies are also given.

## Introduction

According to both epidemiological and clinical results, it has been consistently demonstrated to show a potential causal connection between the consumption of plant-based foods and the reduction of chronic non-communicable diseases, largely attributing to the presence of substantial phytochemicals in plants (1–3). Compared with other production segments including plant growth and agricultural management and post-harvest treatment, the manufacturing operations may show its advantages in the aspects of economic efficiency, direct correlation, and operational accessibility to preserve bioactive compounds of the final products. However, processing approaches may enhance the contents and bioavailability of bioactive components in food. Although conventional thermal processing ensures food safety and the inactivation of deteriorative enzymes and microorganisms of foods, it does not meet the processing requirements of the modern industry of plant-based foods, especially considering the consumers’ demand for fresh-like and health-promoting products. For example, the newly proved tumor-suppressing component indole-3-carbinol, a natural derivative present in cruciferous vegetables, is degraded quickly under various heating conditions, thus resulting in compromised efficiency in its anti-cancer effect (4). The decrease in the content of bioactive compounds after thermal treatments is a general trend found in different plant foods, although there are specific bioactive compounds for which the transformation may fluctuate depending on the types and procedures of heating treatments (5, 6). In addition to molecular changes, specific biological activities of micronutrients may be reduced in light of the impact of processing on the food microstructure and bioaccessibility/bioavailability, as influenced by the re-creation of food matrix microstructure and the changes in the forms of the existing bioactive molecules during heating process (7, 8). Therefore, the application of innovative non-thermal technologies in processing plant-based foods has been extensively studied over the last decade, such as cold plasma, ultrasound, ultra-high pressure (UHP), and pulsed electric fields (PEF).

Ultra-high pressure generally employs water as the pressure-transmitting medium, so it is also called high hydrostatic pressure (HHP), which has shown a wide applicability in the fields of biomedicine (e.g., vaccine production, cancer immunotherapy), biotechnology (e.g., fermentation, enzymatic modification, plant germination and breeding), and food processing (9–11). Particularly, this technology has been recognized as the most successfully developed non-thermal food processing method at industrial level (12). The first commercialized UHP-processed food is the jams launched in Japan in the 1990s (13), mainly pushed by its unique physico-biological effects; that is, UHP-derived stress affects the structure of protein, DNA strands and other metabolism-related functional factors in cell thus inactivating enzymes and microorganisms, but without significantly compromising the organoleptic (e.g., color, flavor) and nutritional characteristics of plant foods. It has been well-recognized that HHP processing (HPP) effects on quality characteristics are influenced by pressure level, holding time and temperature, processing patterns, and sample matrices (composition, texture, etc.). However, increasing researches have demonstrated the limitations of single HHP treatments to inactivate bacterial spores and some resistant enzymes. To achieve that, it is necessary to use HHP in combination with other technologies. In addition, these combined treatments are also used to form new types of food quality and functionalities. For example, HHP can be combined with germination process to increase the biosynthesis and accumulation of bioactive components in plant foods, thus enhancing the nutritional qualities (14–16). Simultaneously, sensory features can be significantly modified after HHP-based combination treatment, thus contributing to develop novel food products with emerging or enhanced features. Regarding grains, combined germination and high pressure treatments have been reported to improve the rheological, pasting, textural, and structural properties of rice flour by adjusting the processing parameters (17, 18).

Correspondingly, this work gives an overview of the major challenges of single high-pressure treatments for completely ensuring food safety and extending the shelf life of the products at the processing conditions which can simultaneously maintain sensorial perception of freshness. After as well as the combination of technologies recently proposed to efficiently improve the applications of HHP.

## Challenges of Single High Hydrostatic Pressure Treatment for Microbial Inactivation

The application of HHP treatments at room temperature destroys the vegetative cells of microorganisms that spoil food products, with minimal changes in most of the nutritional and sensorial food properties. However, this technology shows its limitation in inactivating microbes, particularly Gram-positive microorganisms and spores (19). The baro-resistance of microbes tends to be variable, depending on the type of microorganism, the physiological status and food matrices. Typically, HHP treatments show less efficiencies in inactivating microbes in low-acid juice, where higher resistance of microorganisms to pressure can be found (20). One concerning issue in food safety and preservation, is the re-growth of bacterial populations surviving in the HHP-treated samples during storage. As in the case of Manzanilla Aloreña seasoned table olives, HHP treatments at 300 MPa for 5 min were employed to control yeast populations and avoid their re-growth (21). However, even at the pressure levels of up to 600 or 700 MPa in table olives residual bacterial population could be detected after treatments, probably corresponding to endospore-forming bacteria. HHP affects not only the total number of microorganisms, but also can regulate microbial community structure and diversity in food matrix. Besides, not all populations were affected in the same way. For example, HHP treatments at 600 MPa for 8 min were performed to stabilize brined asparagus for reducing contamination of epiphytic microbiota (22). The relative abundance of *Enterobacteriaceae* was lower than in untreated samples and reduced during storage, while the relative abundance of *Flavobacteriaceae* family (phylum *Bacteroidetes*) was higher in HHP treated samples than in control samples. Substantial evidence has demonstrated that the effect of HHP on microbial diversity is dependent on food matrix types and processing conditions, thus making it hard to assure microbial safety just relying on single HHP treatments.

## Traditional Thermal and High-Pressure Combined Treatments

The major shortcomings of conventional thermal pasteurization for V&F are related to the heat-enhanced reaction kinetics and chemical changes resulting in degradation of bioactive components, nutrients, and deterioration of physicochemical attributes such as tissue browning phenomenon and the production of off-flavor. The use of HHP during thermal treatments allows to decrease the minimal temperature required to ensure microbial and enzymatic inactivation, while maintaining better physicochemical quality of products (23, 24). Nevertheless, for extremely heat resistant ascospores, corresponding processing conditions can cause a more obvious influence on the deterioration of pasteurized fruit products. Alternatively, novel heat-based pasteurization methods, such as ohmic heating and microwave heating or radio frequency heating, have been developed for V&F products. However, although these approaches demonstrate relatively less aggressive heating effects in comparison with conventional thermal processing, some quality aspects would be compromised. For example, ohmic heating could inactivate the vegetable cells of *Bacillus* strains of fermented red pepper with a 99.7% reduction, showing effective microbial inactivation (25). During ohmic heating, the electrical energy is transformed into heat energy thus generating heat internally, reducing the time of processing and increasing the uniformity of the treatment in comparison to conventional thermal treatment in which the heat is transferred by conduction. Unluckily, it remains inevitable to affect thermolabile bioactive components, and to produce other problems such as the electrolysis that could compromise food safety. Specifically, it has been validated in different food matrices that ohmic and conventional heating processes presented similar kinetic and thermodynamic parameters, e.g., degradation rates of anthocyanins and ascorbic acid as well as the patterns of color changes, demonstrating that ohmic heating could produce similar negative effects on the quality of V&F products to conventional heating (26, 27). Similar challenges occur in other novel heating methods. Overall, thermal effects, from whatever sources, tend to negatively influence V&F physicochemical quality and potential bioactivities, and thus high temperature and heating effects should be counterbalanced by adopting non-thermal strategies for microbial inactivation.

## High Hydrostatic Pressure Combined With Non-Thermal Strategies for Ensuring Food Safety

**FIGURE 1:**
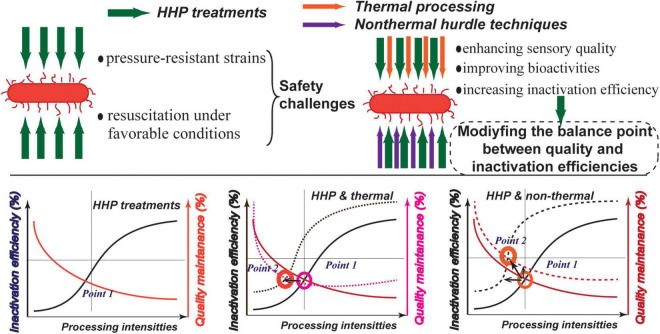
Background for proposing the combination of high hydrostatic pressure (HHP) processing with other hurdle techniques: introduction of HHP-based combination strategies to modify the balance point between quality maintenance and microbial inactivation efficiencies.

High hydrostatic pressure combined with other non-thermal strategies such as ultrasound, essential oils, bacteriocins, PEF, and photocatalysis has been developed as these technologies can enhance the effect on pathogens and deteriorative microorganisms and enzymes while maintaining the physicochemical quality of processed foods. The effect of HHP in combination with other technologies applied in different fruit and vegetable matrices is exemplified in Table 1. The purpose of applying combination modes of HHP is to obtain the optimal point between quality preservation and microbial inactivation (Figure 1).

**TABLE 1:** The effect of high pressure processing (HPP) in combination with other hurdle technologies on the microbiology of different fruit and vegetable matrices.

Combination technologies	Processing parameters	Treatment of products or microbes	Major results regarding microbial inactivation	References
Pressure-assisted thermal processing (PATP)	Samples were preheated at 98°C for 5 min and pressurized at 600 MPa in a vessel set at 90°C for 5 min	pumpkin, butternut squash, pea, beetroot, and purple potato purees	No detectable aerobic and anaerobic microorganisms were found after PATS in all samples, which particularly caused 9 log reduction of *B. amyloliquefaciens* spores	(56) kowsalya
	600 MPa, 10 min, 75°C	Apple juice	3.3 log reduction of ascospores of *Neosartorya fischeri*	(57) kowsalya
	600 MPa, 15 min, 60°C	Tomato pulp	5.7 log reduction of *Bacillus coagulans*	(58) kowsalya
	600 MPa, 10 min, 75°C	Strawberry puree	1.4 log reduction ascospores of *Byssochlamys nivea*	(59) kowsalya
Pressure ohmic thermal sterilization (POTS)	105°C at 600 MPa for 0–5 min, 50 V/cm	green pea puree (pH 6.1), carrot puree (pH 5.0) or tomato juice (pH 4.1)	A reduction of 3.1 and 4.8 log for *B. amyloliquefaciens* and *G. stearothermophilus* spores in tomato juice were obtained, respectively; heat is the primary mechanism of spore inactivation during ohmic heating; POTS which combines pressure, heat, and electrical treatments	(60) kowsalya
HPP + ultrasound	600 MPa, 15 min; thermosonication: 20.20W/mL, 78°C; 60 min	Orange juice	4.4 log reduction of *Alicyclobacillus acidoterrestris*	(33) kowsalya
	450 MPa, 10 min; 25KHz, 70% A, 60 min, 2 W/cm^2^	Apple juice	Complete inactivation of natural microflora	(32) kowsalya
HPP + UV	TiO_2_-UV photocatalysis: 8.45 J/m^2^; 600 MPa, 1 min	Apple juice	7 log reduction of *E. coli* O157:H7, *S. typhimurium, S. cerevisiae, S. aureus*	(37) kowsalya
	TiO_2_-UV photocatalysis: 254 nm, 35 W, 25 mW/cm^2^; 550 MPa, 90 s	*Angelica keiskei* juice	Yeasts and molds, coliform bacteria, Pseudomonas, or *B. cereus* were not detected after combination treatments, but *B. cereus* was recovered after Day 6 of storage	(36) kowsalya
HPP + acidification	400–550 MPa, 1–9 min	*Escherichia coli* O157:H7, *Salmonella enterica*, and *Listeria monocytogenes* in apple juice	Under acidified conditions the prediction models underestimated the magnitude of microbial decrease, implying the role of organic acids in microbial inactivation.	(61) kowsalya
	PATP followed by acidification: HHP (100–800 MPa) and heat treatments (40°C)	*Bacillus coagulans* and *Alicyclobacillus acidoterrestris* in buffer and tomato sauce	*B. coagulans* spores germinated more rapidly at low pH than neutral pH, facilitating a two-stage inactivation treatment wherein germination was first induced by pressure followed by inactivation with heating	(62) kowsalya
HPP + antimicrobial substance	Endolysins: PlyP40, Ply511, or PlyP825; 100–300 MPa, 1–10 min, 30°C	*Listeria monocytogenes*	Synergistic inactivation by endolysins and HHP observed; the pressure enabling the inactivation of Listeria cells became lower	(63) kowsalya
	300 MPa, 20min; essential oils: 200 μl/L	Orange and apple juices	5 log reduction *E. coli* O157:H7 *Listeria monocytogenes*	(41) kowsalya
	500 MPa, 2 min; nisin: 100 IU/mL	Cucumber juice	4 log reduction of total aerobic bacteria and an extended shelf life of 50 days at 4°C.	(40) kowsalya

### Ultrasonication

Ultrasonication (US) technology finds increasing applications in the food industry, involving the aspects of food safety and quality enhancement, particularly expecting the reduction of microbial load simultaneously producing limited detrimental effects on the food quality (28, 29). Substantial ultrasonic processing conditions, including varied intensity waves, single- or multi-frequencies, and different types of ultrasonic generators, have been applied to treat food matrices, and the current results have suggested a relation of the effects on microbial inactivation with the type of microorganism and irradiation conditions (29). The successful use of ultrasound technology for the pasteurization of several fruit juices (e.g., apple, orange, mango) have been validated, and the U.S. Food and Drug Administration (FDA) has recognized it as a technology able to achieve a 5-log microbial reduction (30). Ultrasound-induced microbial inactivation is related to the cavitation bubbles created and the subsequent collapse resulting in local high temperature and high pressure with the formation of free radicals during ultrasonication process. However, a short-time ultrasonic treatment is not robust enough to inactivate microorganisms, but extending the treatment time would compromise nutritional and sensorial properties of V&F products, such as inducing browning phenomenon, and the loss of bioactive compounds. Therefore, ultrasound has recently been applied in combination with other hurdle processes, including UV-C radiation (200–280 nm), and heat (in thermosonication) (30, 31). There are also several studies which have examined the applicability of ultrasound-HHP in V&F processing. The treatment of apple juice with ultrasound, at the frequency of 25 KHz, the amplitude of 70%, and the intensity of 2 W/cm^2^ for 60 min, in combination with HHP (450 MPa for 10 min) achieved complete inactivation of the natural microflora of the product. The cavitation provoked during ultrasound process caused damage to the microorganisms by increasing the temperature and the pressure locally and then, creating shock waves and hydroxyl radicals, which could be the reason of the increase in the lethal effect. The synergic effect avoids the use of higher levels of HHP to achieve the same effect on the microflora (32). In fruit products with low pH (2–4.5), *Alicyclobacillus acidoterrestris* is the most thermal resistant deteriorative microorganism capable to form endospores that could resist thermal pasteurization treatment (80–100°C) and germinate during the shelf-life of the product if the temperature is around 40°C. A pre-treatment at 600 MPa for 15 min followed by thermosonication (20.20 W/mL; 78°C; 60 min) achieved 4.4 log reduction of *Alicyclobacillus acidoterrestris* in orange juice. The effect of thermosonication was enhanced by the germination of the spores induced by the HHP (33).

### Ultraviolet Photocatalysis

Titanium dioxide (TiO_2_)-UV photocatalysis induces the generation of reactive oxygen species (ROS) able to inactivate enzymes and cause oxidative stress in the cellular components of the bacteria. Although TiO_2_ photocatalysis has been developed for a few decades as an antibacterial technology showing large potential for assuring food safety, its extensive applications in the food industries are restricted due to the limited ability of the wide band-gap of TiO_2_ to absorb visible light (34, 35). Therefore, the combination of TiO_2_-UV with other technologies were proposed for enhancing quality characteristics and microbial inactivation. A significant synergistic inactivation effect on *E. coli* O157:H7 was observed in orange fruit juice treated by TiO_2_-UV followed by HHP treatments (300–500 MPa/1 min) (34). Similar phenomenon was observed in freshly squeezed *Angelica keiskei* juice, where yeasts and molds, coliform bacteria, *Pseudomonas*, or *B. cereus* were all inhibited without no colonies detected after a combined processing with TiO_2_-UV and HHP (two cycles of compression at 550 MPa/90 s) (36). Evidence from morphological changes have also demonstrated the presence of synergistic effects of combined treatments (37). It can be concluded that the use of the combined processing with TiO_2_-UV and HHP treatments can provide a non-thermal technological alternatives for ensuring complete disinfection of V&F juices products.

### Antimicrobial Substances

Chemical components with broad antimicrobial spectrum are applied for the disinfection of V&F, including chlorine dioxide, electrolyzed water (EW), ozone, essential oils (EOs), organic acids, and ethanol (38, 39). The use of selected antimicrobial compounds in combination with HHP has been explored, observing significantly enhanced microbial inactivation and extended shelf-life. For example, a bacteriocin in a concentration of 100 UI/ml in combination with an HHP treatment at 500 MPa for 2 min achieved 4 log reduction of total aerobic bacteria in cucumber juice, achieving 50 days of shelf life at 4°C (40). Besides, it was reported that the use of HHP (300 MPa for 20 min) in combination with essential oils (EOs) like (+)- limonene in concentrations of 200 μl/L achieved 5 log reduction of *E. coli* O157:H7 and *L. monocytogenes* in orange and apple juice (41). The combined treatment was used to suppress the regrowth of injured cells during storage. Because of their high volatility, low stability and their impact on organoleptic features of the products, EOs tend to be applied in V&F products through their incorporation into different coatings (e.g., EOs-based nanoemulsion) (42). The synergisms between technologies for the inactivation of pathogenic bacteria have been demonstrated in V&F products by investigating the effects of the combination of nanoencapsulated EOs and multiple non-thermal processing such as UV-C, pulsed light, HHP and γ-irradiation (43, 44). The fabrication of chitosan-based edible nanocoatings is a technology that has recently received substantial attention, due to the unique characteristics of barrier performance of the nanocoating, as well as its mechanical and thermal properties. The newly introduced combination approach, integrating chitosan-based coating with encapsulated EOs followed by HHP treatments (200–400 MPa/5 min), resulted in a more substantial reduction in endogenous *Listeria innocua* load due to the synergism between high pressure-induced membrane structure damage and the antimicrobial activity of the bioactive coating (43).

Electrolyzed water (EW) is used as a rinsing agent for fresh-cut V&F products, often combined with other technologies for hurdle enhancement of antimicrobial efficacy, such as modified atmosphere packaging (MAP), US and HHP (45, 46). EW in combination with mechanical processes (e.g., US, HHP) shows higher efficiencies in controlling microbial loads than individual treatments, as generally observed in EW-US processed fresh-sliced vegetables (47). Relative to US, the application of EW-HHP combined treatment have been less explored although the efficiency of this method has been validated. For example, in case of both fresh-cut carrots and apple, the combined action of slightly acidic EW and HHP was effective on *Escherichia coli* inactivation (48).

On the other hand, in some cases pressure-assisted thermal processing (PATP) is not robust enough to inactivate bacterial endospores so that intensive conditions are still required, e.g., for resistant clostridial endospores where high pressure with up to 120°C was applied to obtain a significant reduction (49). Thus, antimicrobial substances are applied to enhance the inactivation effects of PATP, based on the combined sporicidal effects of heat, pressure and antimicrobial substances in additive or synergistic ways (39, 50). The inactivation effects of antimicrobial substances are species- or strain-specific as affected by the conditions of pressure and temperature as well as the types of antimicrobial compounds. Particularly, the contribution of high pressure or heat to the inactivation relies on the action of modes of antimicrobial components. For example, the nisin addition improved both thermal and PATP-induced inactivation of clostridial endospores relying on synergistic inactivation effect, whereas the other membrane-active antimicrobial agent reutericyclin inhibited the inactivation of the endospores by heat or PATP treatments, attributed to the interaction of the endospore envelope with reutericyclin (49). In addition, regarding small-molecule compounds like EW, organic acids, and ethanol, in PATP treatments they show certain degree of heat stability and simultaneously high pressure cannot disrupt their covalent structure. However, some compounds, including chlorine dioxide, EOs and nisin/bacteriocins, could be pressure or heat sensitive. Therefore, the effects of HHP or thermal HHP treatments on antimicrobial substances needs to be considered particularly at HHP-assisted thermal conditions as thermal or pressure conditions can modify the activities of antimicrobial substances.

The mechanisms responsible for the increased microbial inactivation are generally considered as the result of the transient permeabilization process of antimicrobial substance into intracellular environments, therefore triggering potent intracellular bactericidal action followed by microbial death (38). Similar mechanisms can be found in physical processing such as United States technology. However, not all physical damages induced by non-thermal treatments necessarily lead to significant inactivation of microorganisms, depending on the sub-lethal status and microbial types as well as food matrices. For example, in contrast with HHP, pulsed light did not induce significant synergism of microbial inactivation as revealed by the evaluation of *L. innocua* population (43), which offset the antimicrobial effect of EOs.

### Pulsed Electric Field

It is interestingly to highlight that the simultaneous application of two physical technologies, such as electrically-assisted high pressure (PEF-HHP) processing, has been successfully used for improving food safety (51). This combination, namely PEF-HHP, is different from sonication-mediated HHP, which is implemented only by sequential model (52). When comparing the inactivation efficiencies, individual PEF (20 kV for 1 ms) or HHP treatments (200 MPa/20 min or 400 MPa/5 s) resulted in about one-log reduction; sequential PEF (20 kV for 1 ms)-HHP (200/400 MPa), for both PEF&HHP or HHP&PEF, obtained a two-log reduction while simultaneous PEF-HHP achieved about three log reductions of microbial count. Both additive effects and synergistic effects could be found for *Listeria innocua* inactivation depending on the processing modes of combined PEF and HHP treatments (52, 53), attributed to the action of mechanical and electrical compressive forces.

### CO_2_-Assisted High Hydrostatic Pressure Processing Treatments

In spite that carbon dioxide (CO_2_) is an antimicrobial substance, it is mentioned herein as a separate section because this gas can act as the medium for maintaining the pressure when applied at high pressure conditions, with unique processing features and differential impact on quality properties. CO_2_ demonstrates bacteriostatic and/or fungistatic activities which can be absorbed into food matrix resulting in the acidification and a fluctuation in pH values. CO_2_-assisted HHP treatments, such as high CO_2_ MAP in combination with HHP, sub- or supercritical CO_2_ under pressure conditions (high pressure carbon dioxide, HPCD), as well as carbonation followed by HHP, have been proposed in recent years for liquid samples (e.g., V&F products). Compared with single HHP, CO_2_-assisted HHP treatments have demonstrated their capacity to achieve significantly higher microbial inactivation, related to the explosion, penetration, and acidification effects of carbon dioxide. For example, HPP (400–600 MPa/2 min) in combination with MAP combined was reported to achieve the sublethal injury of over 99.9% of surviving *Listeria innocua* (54).

The high-pressure effects on microbial inactivation can be instable and differential, as revealed by its dependence on pressure levels, holding time, temperature, and microbial species. This kind of dependance can be weakened if extra antimicrobial factors are employed, particularly for pressure-resistant strains. For example, soluble gas stabilization using CO_2_ can be used to significantly inhibit the regrowth of HHP-treated *Listeria innocua* (54). Thus, it is essential to comprehend the key factors and their interaction associated with antimicrobial activities, to adopt right combination patterns and processing parameters, such as the modeling of the gas behavior governing the solubility and diffusivity of CO_2_ in food matrices under HHP conditions (55).

## Concluding Remarks

High hydrostatic pressure processing has shown its great capability to inactivate microorganisms and enzymes and to simultaneously maintain physicochemical stability of food matrices. However, some microorganisms and enzymes are baroresistant. On the other hand, in the last years, several works studied the possibility of enhancing the microbial inactivation with multiple alternative HHP-based combined treatments. Overall, the existing works have mainly demonstrated the feasibility of assuring food safety by using combined strategies. The focus of future research should be pointing to the differences in the molecular mechanisms of microbial response to combined treatments compared with the individual treatments. In addition, several following considerations should be highlighted in the future work:

(i) The overall working mechanisms explaining the enhanced microbial inactivation efficiencies of HHP-based combination technologies are related to the synergistic or additive effects of different inactivation factors (e.g., pressure, temperature, antimicrobial substances). However, the synergies between different technologies in combined treatments remains to be carefully proved. In the cases examining the efficiencies in inhibiting microbial population of HHP-based combined treatments, some experimental designs are not robust to demonstrate the reasonability and necessity of the combination, since combined treatments are only compared with the individual treatments and related analysis of interaction/additive effects are absent or fails to provide satisfied reliability. Synergistic effects and additive effects should be differentiated in terms of inactivation mechanisms and reliable statistical analysis. Therefore, the specific mechanisms for enhanced microbial inactivation should be further explored, in order to justify the need of the combined strategies.

(ii) Combined treatment conditions should be optimized because, at current stage, most researches employ several process parameters to confirm the enhanced microbial inactivation compared with the individual processing approaches.

(iii) The commercialization of the combined technologies may be one of the major limiting factors for employing at industrial applications. The combination or coupling of two processes not only considers the technical feasibility, but also the cost.

(iv) The variability in endogenous microbial flora could be an important factor resulting in differences between kinetic modeling and experimental results. Most of the works reviewed tend to study specific microorganisms in model systems or food matrix, but, in practical, food samples present a lot of microorganisms and the role of microbial interaction could influence the efficiency on the microbial inactivation of proposed novel pasteurization technologies.

(v) Cost analysis and environmental impact; it is noted for combination processes there are two patterns including “simultaneous processes” and “sequential processes.” For example, PEF treatments under high pressure conditions are referred as simultaneous processes; while PEF treatments followed by HHP treatments are referred as successive processes. Generally, additive effects are found in successively applied combination processing modes while synergistic effects in simultaneous modes. However, current research lacks information regarding cost (e.g., energy requirements, treatment time, equipment cost) and environmental impact for both two types of combination processing modes, which should be carefully considered in the future research and industrial application.

## Author Contributions

QX: conceptualization, writing – original draft, writing, review, and editing. GD: writing – original draft, writing, review, and editing. CY: writing, review, and editing. QL, HY, and XC: conceptualization, writing, review, and editing. FB: writing, review, and editing. All authors contributed to the article and approved the submitted version.

## Conflict of Interest

HY and QX were employed by Bright Dairy & Food Co., Ltd. The remaining authors declare that the research was conducted in the absence of any commercial or financial relationships that could be construed as a potential conflict of interest.

## Publisher’s Note

All claims expressed in this article are solely those of the authors and do not necessarily represent those of their affiliated organizations, or those of the publisher, the editors and the reviewers. Any product that may be evaluated in this article, or claim that may be made by its manufacturer, is not guaranteed or endorsed by the publisher.
